# Co-designing a mobile application to reduce self-stigma for people with opioid use disorder during pregnancy and the postpartum period

**DOI:** 10.3389/fpsyt.2025.1607652

**Published:** 2025-09-01

**Authors:** Bailey W. Osweiler, Thue Rammaha, Hannah S. Szlyk, Nathaniel A. Dell, Jushawn Macon, Nicholas C. Jacobson, Casey Burley, Micah Goodman, Patricia A. Cavazos-Rehg, Alex T. Ramsey

**Affiliations:** ^1^ McKelvey School of Engineering, Washington University in St. Louis, St. Louis, MO, United States; ^2^ School of Medicine, Washington University in St. Louis, St. Louis, MO, United States; ^3^ Geisel School of Medicine, Dartmouth College, Hanover, NH, United States; ^4^ Oxford House, Inc., Silver Spring, MD, United States; ^5^ Rissana, LLC, St. Louis, MO, United States

**Keywords:** perinatal, digital intervention, self-stigma, opioid use disorder, ecological momentary assessment and intervention

## Abstract

**Aims:**

Pregnant and postpartum individuals (PPI) face unique challenges to recovery from opioid use disorder (OUD), including stigma from self and others. *Enhearten* is a mobile application featuring an ecological momentary intervention to reduce self-stigma and provide support for perinatal individuals with OUD. This study aimed to refine and test *Enhearten* using the Discover, Design/Build, and Test (DDBT) framework. We hypothesized that DDBT would be associated with increased intervention acceptability, and *Enhearten* would be associated with decreased self-stigma among PPI with OUD.

**Methods:**

In this fully-remote study, participants provided human-centered design feedback in semi-structured interviews at 1-month to guide adaptations. Participants also completed structured questionnaires including validated measures of self-stigma at baseline, 1-month, and 2-month follow-ups and technology acceptance at 1-month and 2-month follow-ups. Paired samples t-tests determined whether differences existed between baseline and 2-month self-stigma and between 1-month and 2-month technology acceptance.

**Results:**

Twenty PPI (40% pregnant, 60% postpartum) representing diverse geographic U.S. regions used *Enhearten*. Qualitative findings highlighted the value of peer support and positive framing but revealed modifiable barriers and a desire for additional features. Human-centered design feedback guided adaptations, including added discussion group features and enhanced relevance of messages. Quantitatively, self-stigma decreased from baseline (M=2.70, SD=0.47) to 2-month (M=2.27, SD=0.61), t(19)=-2.902, p=0.009 (Cohen’s d=0.742). Technology acceptance was high at 1-month and increased by 2-month, t(15)=3.211, p=0.006.

**Conclusions:**

These results support the potential of digital interventions to reduce self-stigma and improve perinatal OUD recovery outcomes. The DDBT framework provides structure to understand lived experiences, adapt rapidly, and evaluate digital intervention efficacy.

## 1 Introduction

The United States (U.S.) continues to face enormous burden from the opioid crisis, with overdose deaths involving opioids surpassing 81,000 in 2022 (1). Approximately 2.5 million Americans have an opioid use disorder (OUD); however, only 1 in 5 receive medication for opioid use disorder (MOUD), with rates even lower among women and Black adults (2). OUD prevalence has increased in recent decades to 6.5 out of every 1000 women having an OUD diagnosis at hospitalization for delivery (3), which is concerning for both mother and baby. Pregnant women with OUD are four times more likely to die before hospital discharge, and these mothers’ risk of opioid overdose deaths increases during the postpartum period (4). Approximately 32,000 infants are born with Neonatal Opioid Withdrawal Syndrome annually in the United States (5), signaling a high rate of babies exposed to opioids *in utero*. These mothers and babies experience intersectional barriers to treatment (6), including criminalization of their conditions, few co-located services (7), and lack of information about managing OUD during pregnancy (8). Although MOUD is the recommended treatment for OUD during pregnancy, these and other barriers interfere with access (9).

Stigmatization of perinatal OUD contributes to these barriers and can come from society, clinicians, or self. “Self-stigma” interferes with recovery outcomes through lower engagement with basic healthcare, reduced uptake of lifesaving MOUD (10), decreased self-efficacy, and increased depression, anxiety, and shame (11, 12). Self-stigma is highly prevalent among pregnant and postpartum individuals (PPI) with OUD (13, 14), including shame, isolation, and fear of losing custody of their child (15); thus, self-stigma reduction is a high priority in this population and is modifiable through an individualized intervention. At least fifteen experimental or quasi-experimental studies published since 2011 investigated treatments for self-stigma, and show promise for self-stigma reduction (16). However, none of these studies focused on PPI or patients with OUD, nor examined self-stigma reduction using contextually appropriate strategies such as digital interventions.

Digital health solutions provide a low-barrier option for individuals who have limited interaction with the healthcare system (17), and research suggests they can reduce self-stigma among individuals with mental health disorders (18). Digital solutions for substance use disorders (SUD) have been shown to be acceptable and effective when tailored to specific populations and rigorously evaluated (19, 20). Despite strong plausibility of digital interventions to address awareness, psychoeducational, attitudinal, and social barriers to OUD recovery, and self-stigma in particular, empirical evidence is needed to support the use of these tools among PPI with OUD (21, 22).

The impact of digital interventions can only be realized if tools are designed to be compelling, intuitive, and embedded in naturalistic environments, a process that is achievable through human-centered design. The Discover, Design/Build, and Test (DDBT) framework is an iterative evaluation process aiming to support human-centered design and to maximize usability of evidence-based interventions in real-world contexts (23). DDBT engages potential end-users throughout the design process, and each phase of the framework generates increased understanding of lived experiences, design needs and preferences, and responses to the intervention. This process ensures the resulting intervention is useful and easy to use, responsive to the intended population needs, and primed to be successfully implemented.

This study aimed to adapt, refine, and test a digital intervention for PPI with OUD using the DDBT framework, with the goal of reducing self-stigma, enhancing self-efficacy to engage in MOUD treatment, and improving mental health. We hypothesize that participants will demonstrate decreased stigma over time and technology acceptance will be higher after the DDBT process. The iterative design and demonstration of both technology acceptance and preliminary efficacy represents a key prerequisite for a large-scale randomized controlled trial to support future commercialization and implementation of the technology.

## 2 Methods

### 2.1 Intervention (*Enhearten*)

*Enhearten* is a mobile health application using twice-daily ecological momentary assessment and intervention (EMA/EMI) features to combat self-stigma among PPI with OUD. The app prompts users to complete a validated questionnaire measuring symptoms of self-stigma. It then presents the app user with one of 120+ tailored videos and one of four tailored messages based on their questionnaire. For example, *Enhearten* includes psychoeducational lessons to challenge negative thinking and increase problem solving skills (24, 25); after prompting the participant to record their feelings of stigma (EMA), a tailored message (EMI) might say, “It looks like what you’re feeling most strongly right now is a sense of other people looking down on you. We all have that feeling sometimes, it’s 100% ok. You’re deserving of recovery no matter what.” In addition to EMA/EMI features, *Enhearten* provides a chat feature between app users, individualized goal setting, and opt-in connection to participating treatment facilities.

### 2.2 Participants

Participants were recruited across all geographic regions of the United States, primarily through SUD treatment programs, recovery residences (i.e., sober living homes), and non-profit organizations such as housing support, job training, family-centered behavioral healthcare, and emergency assistance. Snowball sampling methods were used to supplement recruitment of eligible PPI with OUD. Eligible participants were ≥18 years of age, currently pregnant or within 12-months postpartum, had a self-reported diagnosis of OUD, and had ready access to a mobile phone that enabled the download and use of apps. The study team met via videoconference with each potential participant to conduct eligibility screening and, if eligible, administered electronic informed consent (eConsent) with PPI in REDCap (26, 27).

### 2.3 Study procedure

**Figure 1:**
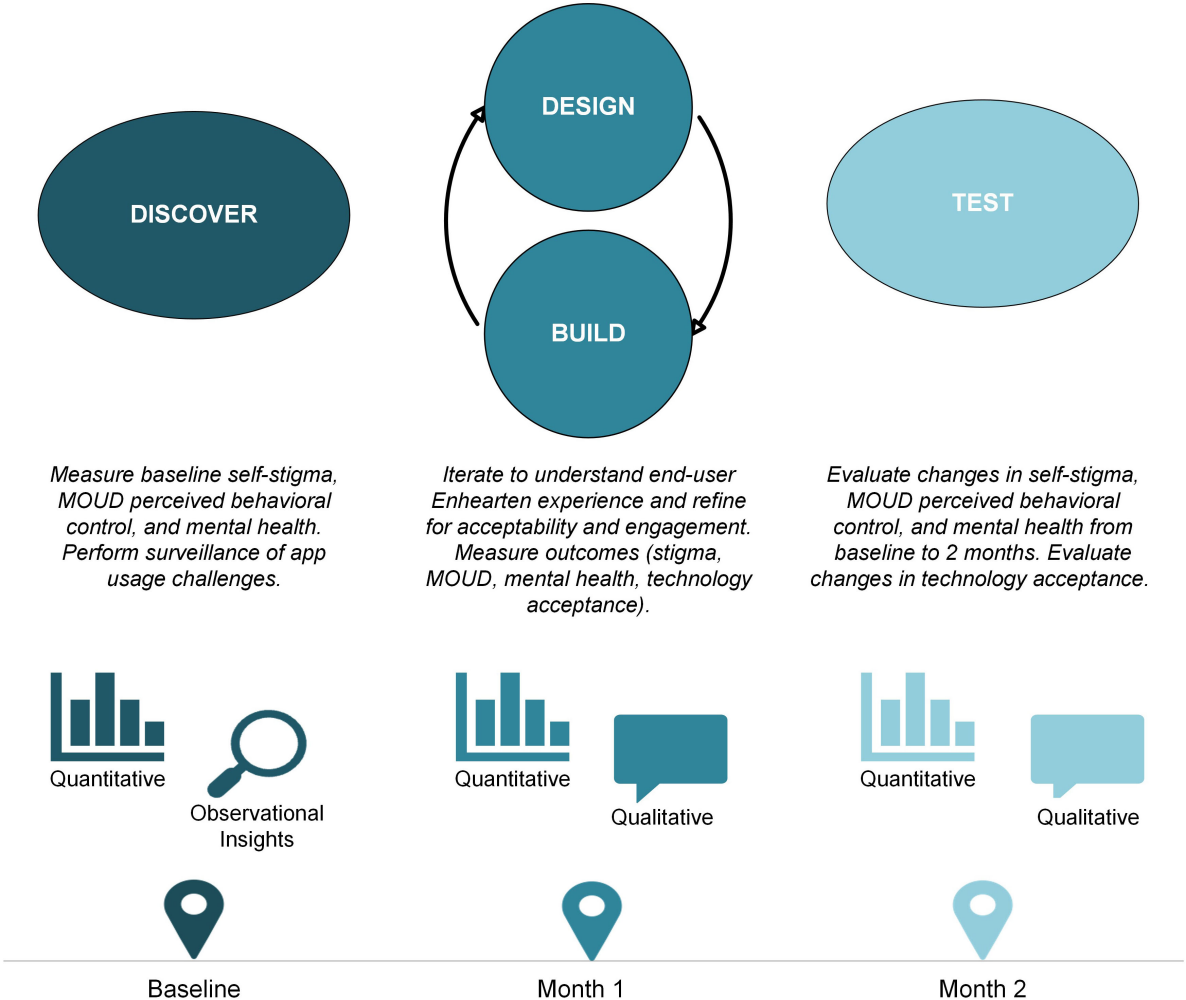
The multiphase Discover, Design/Build, and Test (DDBT) framework as applied to the rapid co-design and evaluation of the *Enhearten* digital intervention.

We followed the DDBT framework to systematically refine and evaluate the digital intervention throughout this study (23). Qualitative measures were assessed via semi-structured interviews, and quantitative measures were assessed via structured questionnaires administered by a trained research member at three time points (baseline, 1-month and 2-month) on videoconference (Zoom) or phone. At baseline, participants completed a questionnaire and then were onboarded to use *Enhearten* at will for a 2-month period. Onboarding tasks included downloading the app, creating and verifying an account, and explaining features. At 1-month, participants completed a questionnaire and provided interview feedback to guide modifications of the application. At 2-month, participants completed a final questionnaire for outcomes assessment and an interview for member checking (**Figure 1**).

#### 2.3.1 Discover

DDBT’s Discover phase includes understanding individuals’ prior experiences, needs, and preferences that may facilitate or hinder an intervention’s usability (23). For this study, the exploratory Discover phase included quantitative baseline questionnaires to understand *Enhearten* adaptation needs in the context of participants’ baseline reports of self-stigma, opioid use, MOUD use, MOUD perceived behavioral control, and mental health symptoms. The Discover phase extended through the first month of *Enhearten* usage for surveillance of onboarding and feedback on initial experiences.

#### 2.3.2 Design/Build

The DDBT Design/Build phase (23) rapidly adapts the intervention using participant feedback. For our study, Design/Build included iterative activities around the 1-month period to collect interim user feedback (qualitative and quantitative). Participants completed a brief structured questionnaire to understand perceived ease of use, usefulness, and intervention acceptability. They completed a semi-structured interview about which digital intervention components were valued and which needed adaptation. Interviews also included a user design component in which researchers and developers screen shared the *Enhearten* app to model simplified examples of user-generated ideas in real-time. The total session lasted a maximum of 1 hour for each participant. We rapidly incorporated salient input to inform additional adaptation and deploy modifications prior to the Test phase.

#### 2.3.3 Test

The final DDBT phase, Test (23), pilots a fully functional application to evaluate usability and usefulness after adaptations, and measure implementation outcomes. For our study, this provided preliminary evaluation of *Enhearten* associations with self-stigma and other outcomes and measured acceptability of the adapted intervention. Participants also engaged in qualitative data collection via member checking by a trained research staff member, aimed at understanding the perceived impact and appropriateness of *Enhearten* adaptations in response to Design/Build phase feedback (28). Member checking increased methodological rigor in the absence of double coding to ensure adaptations were responsive.

### 2.4 Measures

#### 2.4.1 Qualitative

Interview questions focused on perceived benefits of *Enhearten* use, prioritized (and de-prioritized) features, identified barriers and facilitators to app use, and proposed adaptations (**Supplementary Table S2**). Design/Build questions were adaptive such that feedback on a particular feature (e.g., redundancy of EMA questions, responsiveness of EMI, additional community-building features) prompted additional follow-up questions to gain more detailed understanding and explore optimizations.

During the Test phase, questions began broadly to understand participant experiences in using the app and then focused on ways adaptations were responsive or not responsive to their prior feedback.

#### 2.4.2 Quantitative

Demographics: At baseline, we asked participants for their race/ethnicity, age category, perinatal status (pregnant or within 12-months postpartum), education level, employment, and state of residence (**Table 1**).

**Table 1:** Sample characteristics and descriptive statistics (N=20).

Demographic	N (%)
Race/Ethnicity	
White	17 (85%)
Black or African American	3 (15%)
Hispanic	0 (0%)
Asian	0 (0%)
American Indian or Alaska Native	2 (10%)
Native Hawaiian or Pacific Islander	0 (0%)
Other	1 (5%)
Age	
18–30 years	7 (35%)
31–50 years	13 (65%)
Perinatal Status at Baseline	
Pregnant	8 (40%)
Postpartum, childbirth in past 12 months	12 (60%)
Education Level	
High school diploma/GED or less	12 (60%)
Some college, no degree	6 (30%)
Bachelor’s degree	1 (5%)
Master’s degree	1 (5%)
Current Employment	
Yes, full-time	10 (50%)
Yes, part-time	2 (10%)
No, not employed	8 (40%)
State of Residence	
Arizona	2 (10%)
Colorado	1 (5%)
Florida	2 (10%)
Georgia	2 (10%)
Indiana	2 (10%)
Massachusetts	1 (5%)
Missouri	2 (10%)
North Carolina	2 (10%)
Oklahoma	2 (10%)
South Carolina	1 (5%)
Washington	3 (15%)
MOUD use frequency in past 30 days, at baseline	
0	11 (55%)
14	1 (5%)
15	1 (5%)
30	7 (35%)
MOUD used in past 30 days, at baseline	
Methadone	1 (5%)
Buprenorphine/Subutex/Suboxone	8 (40%)
None	11 (55%)

Opioid use and medications for opioid use disorder (MOUD): At baseline, 1-month, and 2-month follow-ups, we assessed frequency of past 30-day opioid use for non-medical purposes and of past 30-day use of MOUD, each with one question on a scale of 0 to 30 days. For those endorsing any past 30-day MOUD use, we asked about types of MOUD used in the past 30 days.

Stigma and self-stigma: At baseline, 1-month, and 2-month, perceived stigma and self-stigma were assessed using the Brief Opioid Stigma Scale (29), measured on a 5-point scale from “strongly disagree” (1) to “strongly agree” (5). Higher scores indicate stronger endorsement of perceived stigma and self-stigma. This measure was selected due to its focus on three key stigma subscales about individuals who use opioids: 1) Aware: stereotype awareness related to perceived public attitudes (e.g., “Most people believe that a person who is addicted to opioids cannot be trusted”), 2) Agree: stereotype agreement related to internalized stigmatizing beliefs (e.g., “I believe that a person who is addicted to opioids cannot be trusted”), and 3) Harm: self-esteem decrement related to the impact of negative stereotypes on self-worth (e.g., “I currently respect myself less because I cannot be trusted due to my addiction to opioids”). Subscales 2 and 3 assessed components of self-stigma, affording the opportunity to understand associations of *Enhearten* with self-stigma (to demonstrate convergent validity) – versus externalized perceptions of public stigma (to demonstrate discriminant validity).

MOUD perceived behavioral control: At baseline, 1-month, and 2-month, we assessed perceived behavioral control to use MOUD using 6 items adapted from Banks and colleagues (30), measured on a 7-point scale from “not at all” (1) to “very” (7), with higher scores indicating higher perceived self-efficacy to use MOUD.

Mental health outcomes: At baseline, 1-month, and 2-month, we assessed self-reported symptoms of depression and anxiety using the 5-item Mental Health Index of the Short-Form Health Survey (31). Items were measured on a 6-point scale from “all of the time” (1) to “none of the time” (6), and all items were coded or reverse-coded with higher scores indicating better mental health over the past month.

Technology acceptance outcomes: At 1-month and 2-month, we used the perceived ease of use (PEU) index and the perceived usefulness (PU) index of the Technology Acceptance Model (TAM) Questionnaire (32) to assess *Enhearten* technology acceptance, and the Acceptability of Intervention Measure (AIM) to understand overall endorsement of the intervention (33). PEU, PU, and AIM each included 4 items measured on a 7-point scale from “totally disagree” (1) to “totally agree” (7). They each represent different components of technology acceptance such as “I find Enhearten easy to use” (PEU) and “using Enhearten helps me with my recovery” (PU), as well as intervention acceptability “Enhearten meets my approval” (AIM).

### 2.5 Analysis

Rapid qualitative analysis methods (34) were used to enable timely adaptations to the technology. Directly following the Discover phase, research team memos were reviewed and prioritized based on urgency (e.g., technical problem with login, nonfunctioning app button) and saliency of responses. Developers made technological repairs in an ongoing and timely fashion shortly after onboarding or as reported to allow each participant the standard 2-month evaluation with a functioning system. In the Design/Build phase, we transcribed qualitative responses verbatim in the interview guide and used a conventional content analysis so themes could emerge organically from the data (35). We also engaged in constant comparison with codes and categories checked throughout data analysis during the Design/Build phase to ensure their continued relevance and appropriateness (36). We identified relevant quotes for each theme and documented corresponding modifications to *Enhearten*. Given the rapid and cyclical nature of analyses and adaptations during Design/Build, some formal conventions in qualitative analysis (e.g., double coding) were not feasible. However, the rigor of qualitative analysis was enhanced via member checking interviews to evaluate responsiveness of modifications to participants’ prior feedback.

All quantitative analyses were completed in SPSS v.29. We first conducted descriptive frequency analyses to understand key demographic indicators of the sample (**Table 1**). We then completed paired samples t-tests to determine whether differences existed between baseline and 2-month follow-up scores on stigma, MOUD perceived behavioral control, and mental health outcomes. We also completed paired samples t-tests to determine whether differences existed between 1-month and 2-month technology acceptance scores after adaptations at 1-month follow-up.

## 3 Results

A total of 20 PPI enrolled, used *Enhearten*, provided feedback, and completed questionnaires. Four participants did not complete the questionnaire at 1-month but were included because they completed the baseline (pre-test) and 2-month (post-test) questionnaire, including our primary outcome of self-stigma.

### 3.1 Discover

#### 3.1.1 Quantitative findings

Most participants identified as White (85%), were 31–50 years old (65%), and postpartum (60%). Participants were from geographically diverse areas, with none reporting current use of opioids for non-medical purposes. About half (45%) were currently taking MOUD, with a larger proportion taking a buprenorphine formulation (40%) compared to methadone (5%) (**Table 1**). Overall, stigma was moderate at baseline (M=2.70; SD=0.47). In particular, the Aware (public stereotype) subscale was high (M=3.96; SD=0.66), the Agree (internalized beliefs) subscale was moderate (M=2.60; SD=0.84), and the Harm (self-esteem decrement) subscale was low (M=1.54; SD=0.74) (**Supplementary Table S1**).

#### 3.1.2 Observational insights from onboarding and early usage

Several key insights emerged during app onboarding and training, and through early-stage spontaneous feedback from participants (e.g. verbal comments during onboarding or user-generated questions via follow-up communications). These insights were instrumental in guiding and prioritizing the focus of Design/Build activities.

Insight #1: EMA is a novel component, with advantages and drawbacks. Participants were intrigued by the EMAs, and this contributed to their interest and engagement in the study. PPI were excited to learn that EMIs would be provided directly in response to their completed EMAs, noting the benefit and novelty of this personalization. At the same time, the approach was not intuitive, leading to many questions about expectations (e.g., response time following EMA prompt) and settings (e.g., how to tailor available time windows for EMA prompts).

Insight #2: PPIs engaged in constructive feedback upon first interactions with the technology. Participants found it engaging to be part of the prospective process to shape the *Enhearten* tool. Several comments foreshadowed themes that more formally emerged in the Design/Build phase. For instance, PPIs were eager to connect with other PPIs in private group chats and cited the importance of using this tool to reinforce their recovery progress. One participant expressed desire for a journal feature that interfaces with the app calendar, to organize a space to reflect on their recovery journey over time.

### 3.2 Design/Build

#### 3.2.1 Qualitative findings

Semi-structured interviews at 1-month follow-up yielded participants’ perspectives on initial use of *Enhearten*. These insights were organized into five key themes (**Table 2**):

1. participants gained comfort and support from *Enhearten*,
2. participants appreciated establishing connections with peers who have similar experiences,
3. participants enjoyed EMI “check-ins” but experienced time and motivation barriers to consistently completing EMAs,
4. participants requested content and support that extends beyond self-stigma, and
5. participants desired positively-framed content and messaging.

**Table 2:** Qualitative themes and corresponding Enhearten adaptations.

#	Theme	Representative quotes	Corresponding modification made to Enhearten
1	Comfort from a personalized digital tool that helps address recovery and mental health needs	“*Many of us are at home, don’t have too much to do, so we like to engage with things that feel useful to us – this app feels like that home base for me.*”	Addition of private, HIPAA-compliant discussion groups for connecting with other PPI, including chat feature for selected topics.
2	Importance of establishing connections among other PPI with related lived experiences	“*Making connections with other pregnant moms with gestational diabetes and in recovery has been so beneficial.*” “*When I get on I see other women supporting other women and we don’t even know each other, so it’s pretty amazing*.”	
3	Strong appreciation for the EMI content, but initial difficulties in awareness or motivation to respond to EMAs	“*I often miss the early morning check-ins.*” “*Just being busy and having 2 kids, I forgot sometimes to take the survey – but it is great to have that twice a day because I recognize that I feel different in the morning versus the evening*.”	App users can now customize the time-of-day settings and the design of their EMI reminders to be more recognizable and inviting.
4	Demand for broader recovery and emotional support content in addition to OUD-related stigma	“*Add more on health and wellness, ways of handling family issues, building community, doing service work – asking what are you doing to support your recovery.*” “*Be hands on with giving pats on the back so that people believe that the app is actually supporting them. We truly need an ultimate recovery app and this has great potential*.”	EMAs now begin by asking app users to reflect on topics that are most on their mind, starting with what is going well, and widening the range of topics that app users can reflect on (e.g., pregnancy/parenting, recovery, mental or physical health, relationships, work-life balance). App users still have the option to reflect on stigma-related questions to receive tailored EMI content.
5	Desire to be presented with and to reflect on positive (instead of negative) developments in their life, recovery, and perceptions of self-stigma	“*The app should focus more on gratitude, appreciation, and* sp*irituality instead of the negative, depressing aspects of addiction.*” “*This is helping me change my perspective – I’m not a victim, not helpless, not due to bad parents, not wallowing in addiction – knowing there are people who can and want to help – now I want to fully open up and share with others so I can help and be helped.*”	

These salient themes were drivers for multiple subsequent modifications proposed to participants at 1-month follow-up and prioritized for completion.

Participants found updates to the EMA/EMI feature to be highly effective in adding variety and relevance to their experiences using the app. One participant stated “I really like the new questions for the check-ins. They are better suited to do daily because now I can answer differently every day. I can think about what is changing and it gives me space to celebrate the good things that are happening which is important when pregnant or recently having a baby”.

In general, there was an appreciative sentiment for the study’s collaborative design and the value of participants feeling engaged through the process; for example, one participant indicated “applying the feedback from participants to the app is very noticeable–I can tell you’re listening. You can scroll through videos and find what you like, interact with other women, and the questions asked in the app are better. The updates are very reflective of the feedback I’ve shared”.

#### 3.2.2 Quantitative findings

Technology Acceptance and Intervention Acceptability results indicated TAM indexes (comprising PEU and PU) and AIM index were high at 1-month and increased by 2-month following the iterative feedback and adaptations (**Table 3**). Given missing data for 4 participants at the 1-month follow-up, paired analyses for these outcomes were reduced (n=16). The TAM scale aggregating PEU and PU of *Enhearten* was rated highly (on a scale from 1 to 7) at the 1-month follow-up (M=6.12, SD=0.68) and increased further by 2-month (M=6.61, SD=0.60), t(15)=3.211, p=0.006. Similarly, the AIM scale measuring *Enhearten* acceptability was rated highly (on a scale from 1 to 7) at 1-month (M=6.25, SD=0.97) and increased further by 2-month (M=6.77, SD=0.54), t(15)=2.534, p=0.023.

**Table 3:** Outcomes pertaining to the effect of Enhearten intervention and iterative design process.

Outcome	Baseline Mean (SD)	1-Month Mean (SD)	2 Month Mean (SD)	Paired Difference: First to Last Measurement Mean (SD)	t	*p*
Technology Acceptance (TAM) and Intervention Acceptability (AIM)						
Overall TAM *(Perceived Ease of Use + Perceived Usefulness)*	–	6.12 (0.68)	6.61 (0.60)	0.49 (0.61)	3.211	0.006
*Perceived Ease of Use e.g. “Learning to use Enhearten is easy for me”*	–	6.58 (0.46)	6.81 (0.52)	0.23 (0.50)	1.861	0.083
*Perceived Usefulness e.g. “I find Enhearten useful”*	–	5.66 (1.05)	6.41 (0.80)	0.75 (1.05)	2.850	0.012
*Intervention Acceptability (AIM) e.g. “Enhearten is appealing to me”*	–	6.25 (0.97)	6.77 (0.54)	0.52 (0.81)	2.534	0.023
Stigma and Self-Stigma (Brief Opioid Stigma Scale)						
Overall	2.70 (0.47)	2.57 (0.54)	2.27 (0.61)	-0.43 (0.66)	-2.902	0.009
*Awareness Subscale e.g. “Most people believe that a person who is addicted to opioids cannot be trusted”*	3.96 (0.66)	3.97 (0.66)	3.63 (1.06)	-0.34 (1.07)	-1.414	0.174
*Agreement Subscale e.g. “I believe that a person who is addicted to opioids cannot be trusted”*	2.60 (0.84)	2.23 (0.81)	1.91 (0.78)	-0.69 (0.85)	-3.600	0.002
*Harm Subscale e.g. “I currently respect myself less because I cannot be trusted due to my addiction to opioids”*	1.54 (0.74)	1.50 (0.80)	1.28 (0.42)	-0.26 (0.67)	-1.750	0.096
Perceived Behavioral Control to Use MOUD						
Overall	5.21 (1.45)	5.79 (1.46)	5.91 (1.49)	0.70 (1.17)	2.690	0.015
Mental Health Index						
Overall	5.20 (0.94)	5.39 (0.87)	5.36 (1.04)	0.16 (0.70)	1.020	0.320

### 3.3 Test

#### 3.3.1 Qualitative findings

Member checking interviews at 2-month follow-up validated the changes made in response to participants’ interview feedback at 1-month follow-up.

Participants greatly appreciated the expanded HIPAA-compliant discussion features created in response to interim 1-month feedback, noting, “I like opening up and knowing it is anonymous. I feel alone sometimes, and it’s nice to share what you’re feeling and get others’ feedback without them knowing who I am.” This was a salient view among several participants, with one recalling a particular instance of using *Enhearten* to offer support and mentorship, sharing, “I remember a time when someone on the app mentioned being in the hospital and how they were treated and I wanted to share my experiences with that person. I remember the time a doctor did not give me a pain medication because I have a history of opioid addiction and wanted to share that experience with others.” Participants were also hopeful that the community within the *Enhearten* app would continue to grow, stating, “If there were more moms on there, there would be a better chance of having others going through the same type of things as me”.

#### 3.3.2 Quantitative findings

Qualitative responses on self-stigma and technology acceptance while using *Enhearten* were reflected in the quantitative data (**Table 3**).

The Brief Opioid Stigma Scale results from baseline (M=2.70, SD=0.47) to 2-month follow-up (M=2.27, SD=0.61) indicated perceived stigma and self-stigma significantly decreased following the *Enhearten* intervention, t(19)=-2.902, p=0.009. *Post-hoc* subscale analyses suggest this change was driven most notably by the decrease in internalized self-stigma on the stereotype Agreement subscale from baseline (M=2.60, SD=0.84) to 2-month (M=1.91, SD=0.78), t(19)=-3.600, p=0.002.

The MOUD Perceived Behavioral Control scale results from baseline (M = 5.21, SD = 1.45) to 2-month (M = 5.91, SD = 1.49) indicated perceived self-efficacy to use MOUD significantly increased following *Enhearten* exposure, t(19)=2.686, p=0.015.

The Mental Health Index results from baseline (M = 5.20, SD = 0.94) to 2-month (M = 5.36, SD = 1.04) did not indicate a significant change in mental health outcomes after the intervention, t(19)=1.021, p=0.320.

## 4 Discussion

The unique and important contributions of this study were finding support for the hypothesis that DDBT would be associated with increased intervention acceptability, and that *Enhearten* would be associated with decreased self-stigma among PPI with OUD. In this mixed-methods study, we piloted a digital intervention for self-stigma reduction with 20 PPI with OUD. Utilizing the DDBT framework provided an iterative structure to receive and incorporate participant feedback rapidly, improving the application design and assessing effectiveness. The intervention, which used EMA/EMI and provided educational and support services for these individuals, demonstrated high technology acceptance that increased after the intervention enhancements resulting from participant feedback. Moreover, the digital solution was associated with stigma reduction (overall and Agreement subscale) from moderate to low and perceived self-efficacy to use MOUD increases after 2 months of app use. These results support the potential of digital stigma reduction tools to improve SUD outcomes.

Participant feedback highlighted several key principles for digital health products for perinatal OUD recovery. While the intervention was associated with a significant decrease in self-stigma, our results indicate individuals require broader supports besides stigma-related resources (Design/Build qualitative finding #4). Participants expressed a desire for comprehensive support addressing multiple dimensions of their lives, including pregnancy, parenting, recovery, mental health, personal relationships, and social determinants of health. Other research shows broad digital tools have higher engagement than focused digital tools (37), suggesting comprehensive support through *Enhearten* may benefit participants. Positive framing is also critical: participants responded most favorably to content emphasizing personal growth, resilience, and hope rather than focusing solely on challenges of OUD (Design/Build qualitative finding #5). This is consistent with prior research on the role of positive framing in SUD recovery (38, 39).

Finally, peer support from other participants was a crucial element of the intervention (Design/Build qualitative finding #2). Participants appreciated the opportunity to connect anonymously and safely with others who shared similar experiences, suggesting practical, emotional support from peers can complement the value of clinical resources. Participants’ appreciation for peer support is encouraging given evidence that peer support may shift stigma (40); although evidence of peer support on OUD recovery outcomes is inconclusive (41), self-stigma is a central feature of *Enhearten* and peer support should be prioritized in future iterations.

### 4.1 Limitations

While the results are promising, this study should be interpreted considering several limitations. The sample size was modest (n=20) and had no control group, although this is in line with extant DDBT studies that apply a human-centered design lens to technology development and early-stage evaluation. Similarly, the study was conducted over a two-month period; *Enhearten*’s long-term effects remain unknown, and the rapid iteration precluded double coding and formal thematic saturation processes. Participants were predominantly White, and none reported active opioid use, limiting generalizability to populations that are actively using opioids for non-medication purposes. This may be a result of recruiting primarily through treatment centers and recovery residences, in which there are recognized racial disparities in treatment, broadly speaking (42), and an expectation of lower rates of active use. Finally, the proportion of individuals receiving MOUD in our study (n=9, 45%) is higher than the national estimate (22%) (2), which may limit generalizability; on the other hand, it is consistent with a recent study finding that 52% of reproductive-age women receiving publicly funded OUD treatment received MOUD (43).

### 4.2 Future work

While our work targeted only self-stigma, stigmatizing messages and interactions from external sources cannot be overlooked; collaborative efforts are needed to address both internal and external stigma. Future research should measure recovery outcomes in addition to stigma and rigorously evaluate *Enhearten’s* efficacy in a randomized control trial (RCT). Such an RCT will require a larger sample size and longer-term follow-up on SUD treatment and recovery outcomes, including among individuals who are actively using opioids. In addition, app usage analytics (e.g., EMA adherence, frequency and duration of app engagement) may reveal more information on technology usability (e.g., which features are used most, changes over time, and associations with outcomes). Finally, investigating moderators to intervention efficacy (e.g., comorbidities, time since opioid use, other treatments) may reveal for whom *Enhearten* is most beneficial and guide further improvements.

## 5 Conclusion

This study used the DDBT framework to collect feedback, iterate rapidly, and evaluate efficacy of *Enhearten*, a digital intervention for self-stigma reduction during perinatal OUD recovery. The adapted tool revealed high technology acceptance and self-stigma reductions after two months. These results support the importance of end-user engagement and feedback in the design process and the potential of digital interventions to reduce self-stigma and improve perinatal OUD recovery outcomes.

## Data Availability

The raw data supporting the conclusions of this article will be made available by the authors, without undue reservation.
