# Supplementary material for: Co-designing a mobile application to reduce self-stigma for people with opioid use disorder during pregnancy and the postpartum period
Source: Front Psychiatry. 2025 Sep 1;16:1607652. doi: 10.3389/fpsyt.2025.1607652 (PMC12434502; doi:10.3389/fpsyt.2025.1607652)
Supplement: Supplementary file 1 [file Table1.docx]

The iterative co-design and test of a digital ecological momentary intervention to reduce perinatal self-stigma involving opioid use disorder

Supplementary Material

# Supplementary Tables

| **Table S1.** Responses to Brief Opioid Stigma Scale, pre and post intervention | | | |
| --- | --- | --- | --- |
| Subscale and item | Mean (SD) at Baseline | Mean (SD) at 2-Month | *p*-value |
| **Aware (public stereotype)** | 3.96 (0.66) | 3.63 (1.06) | 0.174 |
| Most people believe that a person who is addicted to opioids cannot be trusted. | 4.50 (0.76) | 3.90 (1.25) |  |
| Most people believe that a person who is addicted to opioids is dangerous. | 3.90 (0.85) | 3.75 (1.21) |  |
| Most people think that a person who is addicted to opioids is to blame for his or her problems. | 4.30 (0.92) | 3.65 (1.31) |  |
| Most people believe that a person who is addicted to opioids is lazy. | 3.15 (1.35) | 3.20 (1.24) |  |
| **Agree (internalized self-stigma)** | 2.60 (0.84) | 1.91 (0.78) | **0.002** |
| I believe that a person who is addicted to opioids cannot be trusted. | 3.20 (1.24) | 2.00 (1.26) |  |
| I believe that a person who is addicted to opioids is dangerous. | 2.40 (1.19) | 2.00 (1.12) |  |
| I think that a person who is addicted to opioids is to blame for his or her problems. | 2.80 (1.36) | 2.25 (1.37) |  |
| I believe that a person who is addicted to opioids is lazy. | 2.00 (1.12) | 1.40 (0.50) |  |
| **Harm (self-esteem decrement)** | 1.54 (0.74) | 1.28 (0.42) | 0.096 |
| I currently respect myself less because I cannot be trusted due to my addiction to opioids. | 1.65 (1.27) | 1.20 (0.70) |  |
| I currently respect myself less because I am dangerous due to my addiction to opioids. | 1.30 (0.73) | 1.15 (0.67) |  |
| I currently respect myself less because I am to blame for my addiction to opioids. | 1.85 (1.18) | 1.75 (1.12) |  |
| I currently respect myself less because I am lazy due to my addiction to opioids. | 1.35 (0.75) | 1.00 (0.00) |  |
| **Overall Scale** | 2.70 (0.47) | 2.27 (0.61) | **0.009** |

# Supplementary Figures (Box)

**Box S2: Selected prompts from qualitative interviews at 1 month**

**Selected prompts from 1-month semi-structured interview guide**

1. In what ways can the Enhearten app benefit your recovery?
2. What features of the app have you used the most? Why do you think you used those most?
3. What has limited your use of the app? This could include factors in your life or specific to the app.
4. Are there features you think would be beneficial to add to the app? What is missing currently?
5. What would you change about the app, either in terms of content or the way it is organized?
6. These are proposed adaptations based on your and others’ feedback. Do you think these would improve the app or not?
7. Looking specifically at a couple options, which would you prefer we use in the updated app?

**Selected prompts from 2-month member checking**

1. Please share your experiences with using the Enhearten app over the past 2 months.
2. Focusing on your experience within the past month, what changes have you noticed in the app?
3. In what ways were changes responsive to your feedback from last month? In what ways were they not responsive?
